# Trends in Energy Imbalance Gap and Body Weight Status in the Japanese Adult Population: A System Dynamics Approach

**DOI:** 10.2188/jea.JE20190330

**Published:** 2021-05-05

**Authors:** Saeideh Fallah-Fini, Nayu Ikeda, Nobuo Nishi

**Affiliations:** 1 Industrial and Manufacturing Engineering Department, California State Polytechnic University, Pomona, CA, United States; 2 Department of International Health, Johns Hopkins Bloomberg School of Public Health, Johns Hopkins University, Baltimore, MD, United States; 3 National Institute of Health and Nutrition, National Institutes of Biomedical Innovation, Health and Nutrition, Tokyo, Japan

**Keywords:** energy balance, underweight, overweight, obesity, systems science

## Abstract

**Background:**

The double burden of malnutrition is a growing public health problem in Japan. We estimated the dynamics of the energy imbalance gap (EIG) (average daily difference between energy intake and expenditure) to explain trends in the prevalence of underweight, overweight, and obese Japanese adults.

**Methods:**

We used individual-level data on body height and weight from the National Health and Nutrition Surveys from 1975 to 2015. We calibrated a validated system dynamics model to estimate the EIG for Japanese adults aged 20 to 74 years by survey year, sex, and weight status classified by the body mass index (BMI).

**Results:**

The overall EIG for men increased from 2.3 kcal/day in 1975 to 4.7 kcal/day in 1987 and then decreased to 2.3 kcal/day in 2015. The overall EIG for women consistently decreased from 4.3 kcal/day in 1975 to −0.5 kcal/day in 2015. By BMI class, the EIG for men with a BMI of <30 kg/m^2^ began to decrease around 1990, indicating a deceleration in the prevalence of overweight and obese men. The EIG consistently decreased for women with a BMI of <25 kg/m^2^ and reached negative values from the late 2000s to early 2010s, indicating a gradual decrease in the prevalence of overweight and obese women.

**Conclusions:**

The dynamics of the EIG were different across sex and weight groups. Public health interventions should target a further decrease in the EIG for normal-weight, overweight, and obese men and a stop in the decreasing trends of the EIG in underweight and normal-weight women.

## INTRODUCTION

The double burden of malnutrition is an important public health issue in Japan. In 2017, the prevalence of an underweight status in Japanese adults aged 20 years and older was 4% among men and 10% among women, and the prevalence of an overweight and obese status was 31% among men and 22% among women.^1^ An elevated risk of all-cause mortality associated with a high and low body mass index (BMI) has been observed in cohort studies in Japan.^2^^,^^3^ The national health promotion titled Health Japan 21 (the second term) set a target to reduce the percentages of underweight, overweight, and obese adults through lifestyle modification and improved social environments for extension of healthy life expectancy and reduction of health disparities by the year 2022.^4^

Weight gain and loss originate from an imbalance between energy intake and energy expenditure, quantified by the energy imbalance gap (EIG). Understanding the dynamics of the EIG can help explain the driving forces behind the shifts in the distribution of body weight and estimate the magnitude of changes in energy intake and/or physical activity required to reverse such shifts. In previous studies, a system dynamics model was applied to estimate the dynamics of the EIG from anthropometric data of national cross-sectional surveys in the United States and New Zealand, and these studies showed that the trends of the EIG differed by sex, ethnicity, and BMI class.^5^^,^^6^ In the present study, we adopted a validated system dynamics model to estimate the dynamics of the EIG and thus understand the trends in the body weight status in the general adult population in Japan during the past 4 decades.

## METHODS

### Data source and study subjects

We obtained individual-level data from the National Nutrition Surveys of 1975 to 2002 and the National Health and Nutrition Surveys of 2003 to 2015. These were annual cross-sectional household interview and examination surveys conducted on nationally representative samples every November by Japan’s Ministry of Health, Labour and Welfare. The surveys were designed to collect basic data on the health, nutrition, and lifestyle of adults and children for comprehensive promotion of the nation’s health. The methods of the National Nutrition Survey and National Health and Nutrition Survey have been described in detail elsewhere.^7^^,^^8^ At a physical examination site, public health nurses measured survey participants’ standing height barefoot with a stadiometer to the nearest 0.1 cm and weight in light clothing to the nearest 0.1 kg. We calculated the BMI as the weight in kilograms divided by the square of the height in meters. The subjects of our analysis were individuals aged 20 to 74 years. We excluded pregnant women and counted the number of participants aged 19 years and 20 to 74 years separately by survey year, sex, and BMI class (15.0–17.9, 18.0–19.9, 20.0–22.9, 23.0–24.9, 25.0–27.9, 28.0–29.9, 30.0–32.9, 33.0–34.9, 35.0–37.9, 38.0–39.9, 40.0–45.9, 46.0–53.9, 54.0–61.9, and 62.0–68.0 kg/m^2^). We also calculated the mean height in centimeters of subjects aged 20 to 74 years by survey year and sex. We obtained published tables on the number of all-cause deaths of subjects aged 20 to 74 years by sex from Vital Statistics^1^ and of individuals in the Japanese population aged 19 years and 20 to 74 years by sex from the Population Census.^9^

Under the Statistics Act,^10^ the Ministry of Health, Labour and Welfare anonymized individual-level data collected from the National Health and Nutrition Survey before providing us with the datasets. No ethical review was therefore necessary in accordance with the Ethical Guidelines for Medical and Health Research Involving Human Subjects.^11^ The data were processed by the researchers at the International Center for Nutrition and Information (part of the National Institute of Health and Nutrition within the National Institutes of Biomedical Innovation, Health, and Nutrition) to provide the population-level measures that were later used for calibration of the simulation model.

### Simulation analysis

We defined the EIG as the average daily difference between energy intake and expenditure.^5^^,^^6^ The EIG takes positive values for weight gain on average, negative values for weight loss, and a value of 0 for no change. We conducted the simulation analysis separately by sex because the distributions of the BMI and their trends were different between the sexes. We applied a population-level system dynamics model to relate changes in the distribution of the BMI to changes in the EIG. The concepts and methodologies of the model have been detailed elsewhere.^12^ In the present analysis, we first divided BMI into 14 distinct partitions (classes), as defined in the previous section, each one containing members of the subpopulation whose BMI fell within the interval of BMI values associated with that stock. A hypothetical representative individual was assigned to each stock to connect the micro-level dynamics of individual weight gain and loss to the macro-level distribution of the population BMI. Representative individuals were assumed to have the average of the BMI range that corresponded to their stocks. Each stock was connected to neighboring stocks by flows of population at the rate that was a function of the BMI change associated with representative individuals. The dynamics of BMI change for representative individuals over time were modeled using the Hall et al^14^ model of body weight change. The BMI change of representative individuals was then related to the imbalance between energy intake and energy expenditure of individuals, represented by the EIG. As representative individuals gained or lost weight, they pushed a percentage of the population in their corresponding stocks to the neighboring stocks. Consequently, by repeating these steps throughout the survey years, we modeled the rate of shift in the BMI distribution and captured the dynamics of the population BMI resulting from the EIG.

To ensure that the system dynamics model was representative of the Japanese adult population, we calculated the rate of transition from adolescence (age of 19 years) into adulthood (age of 20–74 years) by BMI class in each survey year and interpolated the transition rates when data were missing. We also incorporated all-cause mortality rates at the age of 20 to 74 years by BMI class in the model. To calculate all-cause mortality rates by BMI class, we considered the differential mortality between a very low BMI and very high BMI using mortality adjustment curves.^13^

We modeled the EIG associated with the representative individual of the k^th^ BMI class (k = 1, 2, …, 13, 14) at year *t* (1975, 1976, …, 2014, 2015) as a product of the energy required for normal activity and maintenance of the body, ${\text{E}}_{\text{k}}{}^{*}$, and an energy gap multiplier, $\mu_{\text{kt}}$ (Equation 1).

$${EIG}_{\text{kt}}=I_{\text{kt}}-E_{\text{k}}{}^{*}=E_{\text{k}}{}^{*}\times \mu_{\text{kt}}$$ (1)

We used a model of body weight regulation to calculate ${\text{E}}_{\text{k}}{}^{*}$.^14^ We then added EIG_kt_ to ${\text{E}}_{\text{k}}{}^{*}$ to calculate energy intake for each representative individual, I_kt_. We defined $\mu_{\text{kt}}$ as a function of time (survey years), the BMI of representative individuals, and the interaction between time and the BMI (Equation 2).

$$\mu_{\text{kt}}={\text{Time effect}}_{\text{t}}+{\text{BMI effect}}_{\text{k}}+{\text{Interaction effect}}_{\text{kt}}$$ (2)

$${\text{Time effect}}_{\text{t}}=\beta_{1}+\beta_{2}\times {\text{Time}}_{\text{t}}+\beta_{3}\times {\text{Time}}_{\text{t}}{}^{2}+\beta_{4}\times {\text{Time}}_{\text{t}}{}^{3}$$

$${\text{BMI effect}}_{\text{k}}=\beta_{5}\times {BMI}_{\text{k}}+\beta_{6}\times ({BMI}_{\text{k}}{)}^{\beta 7}$$

$${\text{Interaction effect}}_{\text{kt}}=\beta_{8}\times {\text{Time}}_{\text{t}}\times {BMI}_{\text{k}}$$

We used the maximum likelihood method to estimate these eight beta parameters such that the BMI distributions generated by the model were as close as possible to the observed BMI distributions obtained from the survey samples. We initialized the model using the BMI distributions observed in the 1975 survey. We then simulated the model through 2015 and calculated the likelihood of observing the BMI distributions in the survey samples. The overall log-likelihood function summed up the logarithm of likelihood values across survey years.

In addition to estimating the EIG associated with each BMI class separately over time for each sex, we also calculated the overall EIG for each sex by averaging the EIG across different BMI classes weighted by the population percentage in those classes. Moreover, we calculated the overall EIG for five weight categories (ie, underweight, normal weight, overweight, obese, and severely obese) for each sex by averaging the EIG across different BMI classes associated with each weight category weighted by the population percentage in those BMI classes.

We defined the maintenance energy gap as a measure of the increased energy intake needed to maintain a higher average body weight compared with the initial average of the body weight in 1975.^6^ We calculated the maintenance energy gap for each sex by first calculating the average daily energy expenditure for each year. Next, we subtracted the average daily energy expenditure in 1975 from the average daily energy expenditure in each survey year to find the increased energy intake needed to maintain higher average body weights over time. The average daily energy expenditure in each year was calculated by averaging the daily energy expenditure of representative individuals across BMI classes weighted by the population percentage in those BMI classes.

To validate our results, we used the one-sample Kolmogorov–Smirnov test to examine whether the BMI distribution simulated by the system dynamics model was different from the BMI distribution observed in the surveys. We conducted the data processing by Stata version 14 (StataCorp, College Station, TX, USA) and all simulations and optimizations by Vensim™ (Ventana Systems, Inc., Harvard, MA, USA).^15^

## RESULTS

**Figure 1.**
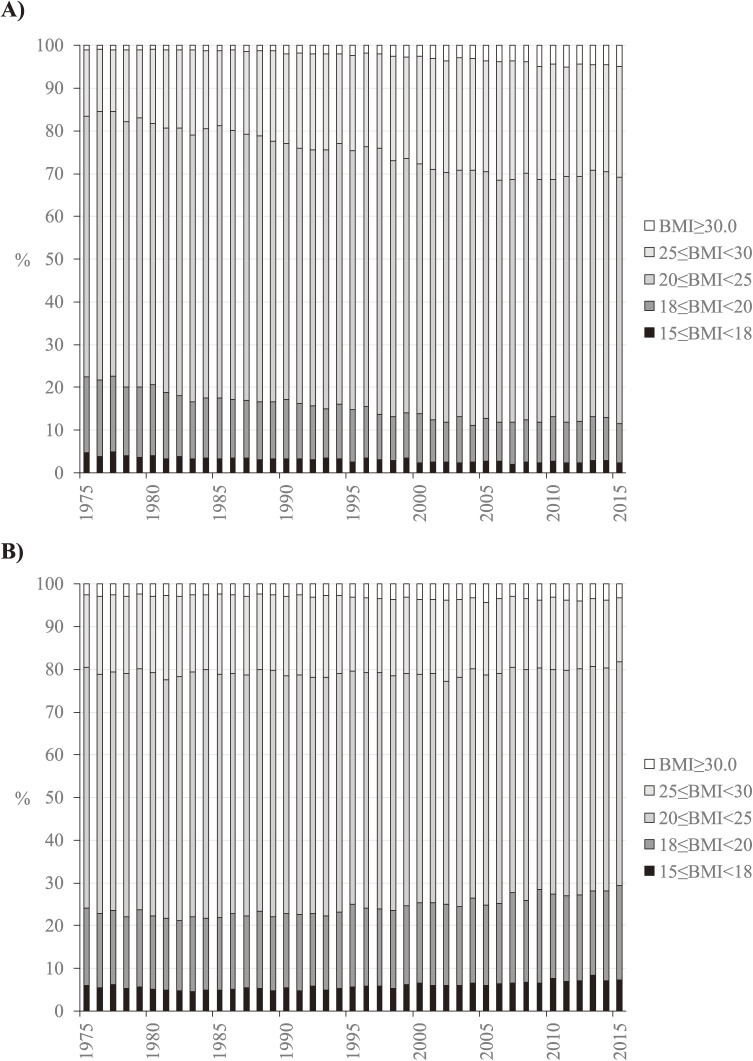
Distribution of BMI in Japanese (A) men and (B) women aged 20 to 74 years, 1975 to 2015. BMI, body mass index

Figure 1 shows the distribution of BMI classes observed in survey participants aged 20 to 74 years from 1975 to 2015 by sex. The prevalence of overweight and obese men (BMI of ≥25.0 kg/m^2^) increased from 16% in 1975 to 31% in 2006 and remained stable thereafter, and the prevalence of underweight men (BMI of 15.0–17.9 kg/m^2^) decreased from 5% in 1975 to 2% in 2000 and remained stable thereafter (Figure 1A). The prevalence of overweight and obese women remained constant at 18% to 23% across the study period, and the prevalence of underweight women was 5% to 6% from 1975 to 2006 and slightly increased to 7% to 8% in 2007 and thereafter (Figure 1B). The simulation model’s beta parameters associated with $\mu_{\text{kt}}$ were estimated for each sex through calibration and are shown in Table 1.

**Table 1.** Model parameters of the energy gap multiplier estimated through calibration, by sex, for Japanese adults aged 20 to 74 years, 1975 to 2015

Parameters	Men	Women
Time effect		
β1	0.042	0.180
β2	0.002	−0.019
β3	−0.017	0.016
β4	0.008	−0.009
Body mass index effect		
β5	0.036	−0.011
β6	−0.080	−0.174
β7	0.389	0.004
Interaction effect		
β8	0.019	0.028

### EIG for men

**Figure 2.**
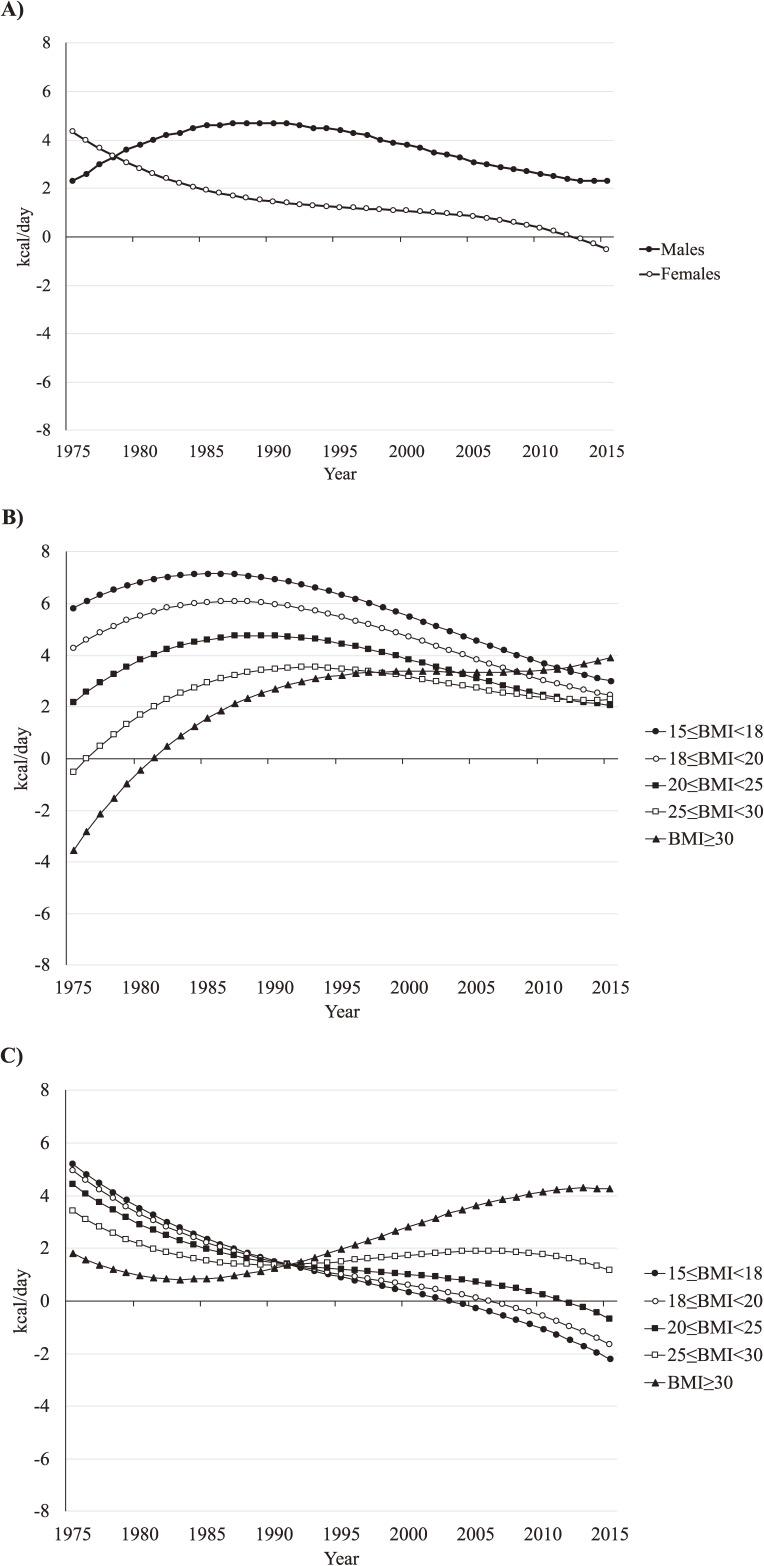
Estimated energy imbalance gap (kcal/day) for Japanese adults aged 20 to 74 years, 1975 to 2015, by (A) sex and by BMI class in (B) men and (C) women. BMI, body mass index.

The overall EIG for men increased from 2.3 kcal/day in 1975 to 4.7 kcal/day in 1987 and then began to decrease in 1992, returning to 2.3 kcal/day in 2013 (Figure 2A). By BMI class, the EIG for men ranged from −3.5 kcal/day for a BMI of ≥30.0 kg/m^2^ to 4.6 kcal/day for a BMI of 15.0 to 17.9 kg/m^2^ in 1975 (Figure 2B). The EIG increased for men with a BMI of 15.0 to 17.9 and 18.0 to 19.9 kg/m^2^, peaking at 7.2 and 6.1 kcal/day, respectively, in the mid to late 1980s and decreased to 3.0 and 2.5 kcal/day, respectively, in 2015. A similar trend in the EIG was observed for men with a BMI of 20.0 to 24.9 and 25.0 to 29.9 kg/m^2^, peaking at 4.8 and 3.5 kcal/day, respectively, in the late 1980s to early 1990s. The EIG for a BMI of ≥30.0 kg/m^2^ continued increasing from 1975 to 2015.

### EIG for women

The overall EIG for women continued to decrease from 4.3 kcal/day in 1975 to −0.5 kcal/day in 2015 (Figure 2A). The EIG for women by BMI class in 1975 ranged from 1.8 kcal/day for a BMI of ≥30.0 kg/m^2^ to 5.2 kcal/day for a BMI of 15.0 to 17.9 kg/m^2^ (Figure 2C). The EIG for women with a BMI of ≥30.0 kg/m^2^ decreased slightly until the mid 1980s followed by a continuous increase throughout the rest of the study period. The EIG then continued to decrease for women with a BMI of <25.0 kg/m^2^, reaching a negative value from the early 2000s to early 2010s. The EIG for women with a BMI of 25.0 to 29.9 kg/m^2^ slightly increased in the 1990s, plateauing or beginning to decrease from the late 2000s to early 2010s.

### Maintenance energy gap

Table 2 shows the estimated average daily energy expenditure and maintenance energy gap by sex and survey year. For men, the maintenance energy gap increased over time to 81 kcal/day in 2015. This indicates that on average, men consumed and expended 80 kcal/day more in 2015 to sustain their increased body weight compared with the energy they had consumed and expended in 1975. For women, the maintenance energy gap increased to 8 kcal/day in the early 1980s and then began to decrease in the late 2000s, reaching −5 kcal/day in 2015.

**Table 2.** Estimated average daily energy expenditure and MEG in Japanese adults aged 20 to 74 years, by sex and survey year

Year	Men		Women	
	Average daily energy expenditure	MEG (kcal)	Average daily energy expenditure	MEG (kcal)
1975	1,897		1,513	
1976	1,898	1	1,515	2
1977	1,900	3	1,516	3
1978	1,903	5	1,518	5
1979	1,905	8	1,518	6
1980	1,908	10	1,519	7
1981	1,910	13	1,520	7
1982	1,913	16	1,520	8
1983	1,916	19	1,521	8
1984	1,919	22	1,521	8
1985	1,922	25	1,521	8
1986	1,925	28	1,520	8
1987	1,927	30	1,520	7
1988	1,930	33	1,520	7
1989	1,933	36	1,520	7
1990	1,936	39	1,519	6
1991	1,939	42	1,519	6
1992	1,941	44	1,519	6
1993	1,944	47	1,518	5
1994	1,946	49	1,517	5
1995	1,949	51	1,517	4
1996	1,951	54	1,516	4
1997	1,953	56	1,516	3
1998	1,956	58	1,516	3
1999	1,958	60	1,515	2
2000	1,959	62	1,515	2
2001	1,961	64	1,514	2
2002	1,963	66	1,514	2
2003	1,965	68	1,514	2
2004	1,966	69	1,514	1
2005	1,968	70	1,514	1
2006	1,970	72	1,513	0
2007	1,971	74	1,513	−0
2008	1,972	75	1,512	−0
2009	1,973	76	1,512	−1
2010	1,974	77	1,512	−1
2011	1,975	78	1,511	−1
2012	1,976	79	1,511	−2
2013	1,976	79	1,510	−3
2014	1,977	80	1,509	−4
2015	1,978	81	1,508	−5

MEG, maintenance energy gap.

### Model validation

**Figure 3.**
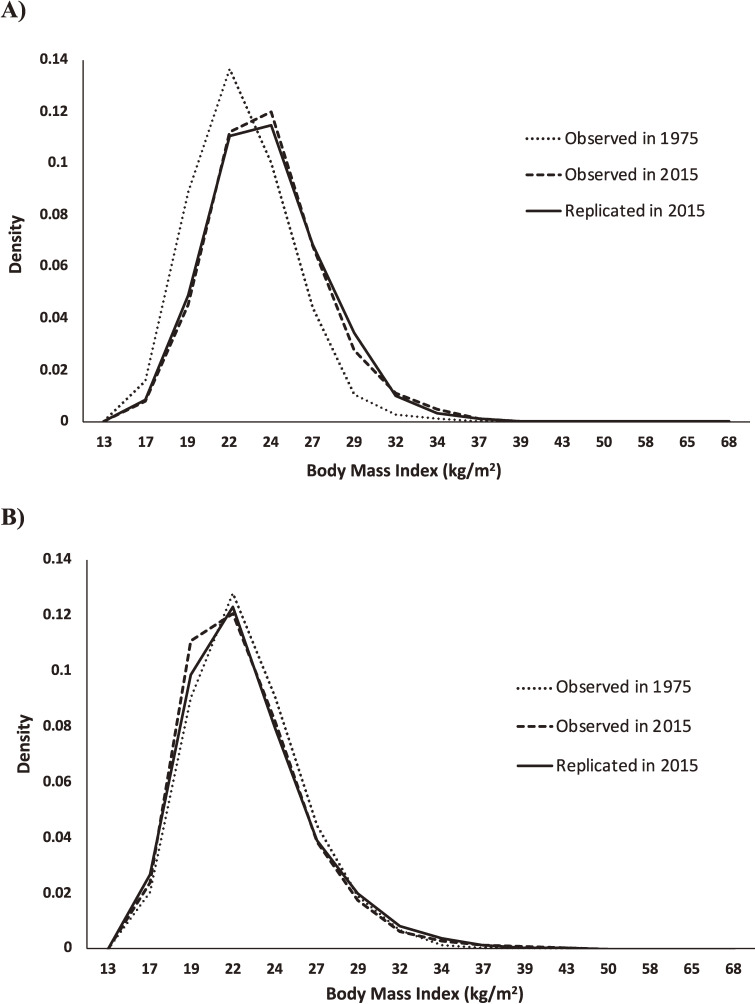
Observed body mass index distributions from the Japan National Health and Nutrition Survey in 1975 and 2015 and the distribution in 2015 replicated by the system dynamics model for Japanese (A) men and (B) women aged 20 to 74 years

Among the 41 survey years, the Kolmogorov–Smirnov test rejected the null hypothesis of no difference between the observed BMI distribution and the simulated BMI distribution in 5 survey years in men and 2 survey years in women (Table 3). Figure 3 shows the observed BMI distributions in 1975 and 2015 and the simulated BMI distribution in 2015. The simulation model replicated the shift in the distribution of the BMI to the right in men and slightly to the left in women from 1975 to 2015.

**Table 3.** Test statistics (critical values) of the Kolmogorov–Smirnov test on the difference between body mass index distributions observed in the survey and those simulated by the system dynamics model

Year	Men		Women	
1975	0.000	(0.021)	0.000	(0.017)
1976	0.010	(0.019)	0.014	(0.016)
1977	0.020	(0.021)	0.005	(0.018)
1978	0.011	(0.020)	0.016	(0.017)
1979	0.005	(0.020)	0.013	(0.017)
1980	0.010	(0.019)	0.008	(0.016)
1981	0.016	(0.022)	0.016	(0.018)
1982	0.016	(0.020)	0.008	(0.017)
1983	0.028^a^	(0.021)	0.011	(0.017)
1984	0.004	(0.021)	0.010	(0.018)
1985	0.007	(0.019)	0.007	(0.017)
1986	0.006	(0.020)	0.009	(0.017)
1987	0.006	(0.021)	0.009	(0.018)
1988	0.005	(0.020)	0.017	(0.018)
1989	0.007	(0.022)	0.012	(0.019)
1990	0.014	(0.021)	0.009	(0.019)
1991	0.015	(0.021)	0.014	(0.019)
1992	0.009	(0.021)	0.019	(0.019)
1993	0.022^a^	(0.021)	0.012	(0.019)
1994	0.016	(0.022)	0.007	(0.020)
1995	0.008	(0.022)	0.019	(0.020)
1996	0.021	(0.022)	0.008	(0.020)
1997	0.020	(0.022)	0.005	(0.020)
1998	0.027^a^	(0.021)	0.011	(0.020)
1999	0.011	(0.024)	0.019	(0.021)
2000	0.014	(0.023)	0.012	(0.021)
2001	0.029^a^	(0.024)	0.010	(0.021)
2002	0.024	(0.025)	0.026^a^	(0.022)
2003	0.016	(0.025)	0.023^a^	(0.022)
2004	0.015	(0.028)	0.017	(0.025)
2005	0.007	(0.028)	0.016	(0.026)
2006	0.038^a^	(0.027)	0.013	(0.025)
2007	0.021	(0.027)	0.021	(0.025)
2008	0.010	(0.027)	0.007	(0.024)
2009	0.014	(0.027)	0.025	(0.025)
2010	0.020	(0.028)	0.011	(0.026)
2011	0.014	(0.029)	0.006	(0.027)
2012	0.010	(0.015)	0.006	(0.014)
2013	0.019	(0.028)	0.011	(0.026)
2014	0.020	(0.029)	0.010	(0.026)
2015	0.008	(0.030)	0.016	(0.027)

^a^Test statistic > Critical value.

## DISCUSSION

We applied a multidisciplinary systems science approach to connect the dynamics of individuals’ body weight gain and loss to the dynamics of the population-level distribution of the BMI. By calibrating the system dynamics model by the nationally representative cross-sectional data, we estimated the dynamics of the EIG that underlie the trends in the prevalence of underweight, overweight, and obese Japanese adults during the past four decades.

Our results show that the dynamics of the EIG were different between men and women. In the late 1970s, the overall EIG was estimated to be positive for both men and women. The overall EIG for men increased over time, peaking in the late 1980s and then gradually decreased until 2015. The overall EIG was still positive in 2015, suggesting that the prevalence of an overweight and obese status will continue to increase in Japanese men, albeit at a slower rate. For women, the overall EIG consistently decreased during the past four decades and began to show a negative value in the 2010s, indicating that the prevalence of an obese and overweight status has begun to decrease among Japanese women.

As expected, the estimated trends of EIGs by BMI class justified the observed trends in the distribution of BMI for both men and women. For example, the positive EIG values for underweight and normal-weight men over time indicate a gradual shift of this population to the overweight class, justifying the increase in the prevalence of an overweight status toward 2015. The positive EIG values for overweight and obese men also indicate an increasing trend in the prevalence of obesity over time. Similarly, underweight women began showing negative EIG values around the mid 2000s. This indicates that a majority of the underweight female population was losing weight. A similar trend was observed for normal-weight women, indicating that a majority of this population was losing weight to move toward the underweight class and justifying the slight increase in the prevalence of an underweight status toward 2015.

Public health interventions mainly target the EIG as the key factor to reduce the prevalence of an underweight, overweight, and obese status among the population.^16^ Our results imply that to reduce the prevalence of overweight and obese adults, public health interventions should focus on reducing the EIG of the overweight and obese population to negative values, so that these individuals begin to lose weight on average. Moreover, to reduce the prevalence of underweight adults, the EIG for this weight group should be increased to positive values so that they begin to gain weight on average.

A similar analysis was performed to estimate the EIG underlying the rise in the prevalence of overweight and obese adults in New Zealand^5^ from 1988 to 2014 and in the United States from 1971 to 2010.^6^ In New Zealand, the EIG was estimated for European/other, Māori, Asian, and Pacific men and women. The overall estimated EIG for European/other women increased over time with a peak around early 1990, a decrease until 1999, a subsequent slight increase, and a peak around 2009. For New Zealand European/other men, the overall estimated EIG peaked about a decade later, around 2000. The overall EIG for Māori women increased until 2002 and subsequently decreased. For Māori men, the overall EIG slightly decreased until 1994 and then peaked around 2003. The overall EIG for Pacific women increased over time, with a peak around 2000 followed by a spike again around 2010. For Pacific men, the overall EIG continuously increased during the past three decades. For Asian men and women, the overall EIG increased over time, peaking around 2006. The EIG was negative in 2014/2015 only for Māori and Asian men and women.

In the United States, the EIG was estimated for non-Hispanic white, non-Hispanic black, and Mexican American men and women. For both non-Hispanic whites and non-Hispanic blacks, the overall estimated EIG was higher in the late 1980s to early 1990s than in the early to late 1970s. The increase in the magnitude of the EIG continued until around 2002 in these two subpopulations followed by a gradual decrease. The magnitude of the decrease in the EIG was larger in non-Hispanic whites than non-Hispanic blacks. Mexican Americans showed an increase in the estimated EIG from the late 1980s to 2010. Overall, non-Hispanic black and Mexican American women showed a larger EIG than men. However, non-Hispanic white men had a larger energy surplus than women from the late 1980s until 2010. None of the subpopulations in the United States showed a negative EIG in 2010.

Our results suggest two areas for future research in public health and epidemiology. First, the effects of socio-environmental factors on the EIG should be explored over time. The EIG is driven by major environmental, economic, and social trends.^16^^,^^17^ It is important to understand the roles of these socio-environmental factors in determining the body weight status of the adult population in Japan during the past 4 decades. Another important direction of research is to assess the impact of public health interventions on different subpopulations based on their sex and BMI. This is feasible by evaluating the effects of interventions on the magnitude of the EIG of individuals, which can be translated into the shifts in the distribution of the BMI and consequently the changes in the prevalence of an underweight, overweight, and obese status. Thus, we can identify interventions that may have the greatest potential impact on prevention of an underweight, overweight, and obese status.

The present study has several limitations. First, we did not construct the system dynamics model by age group, although the distribution of the BMI varies with age. For example, the prevalence of overweight and obese women in 2015 ranged from 10% at the age of 20 to 29 years to 22% at the age of 60 to 69 years.^18^ Second, we did not consider changes in the population’s age structure over time, which may have resulted in bias toward older people in recent years. Third, our model does not account for demographic changes due to immigration and emigration. Finally, use of the BMI as an anthropometric index of an overweight and/or obese status has been shown to introduce bias from misclassification. BMI estimation for individuals who are muscular or have little muscle may not be accurate and can cause overestimation or underestimation of their BMI class.

In conclusion, the dynamics of the EIG differed across sex and weight groups in the general adult population of Japan during the past four decades. This indicates the presence of health disparities by sex and weight groups that may be affected differently by the environment or may respond differently to various interventions. To reduce the prevalence of overweight and obese adults, public health interventions should focus on reducing the EIG of the overweight and obese population to negative values so that these individuals begin to lose weight on average. Moreover, to reduce the prevalence of underweight adults, the EIG for this weight group should increase to positive values so that they begin to gain weight on average.
